# Feasibility of a No-Implant Approach to Interatrial Shunts: Preclinical and Early Clinical Studies

**DOI:** 10.1016/j.shj.2022.100078

**Published:** 2022-08-09

**Authors:** Colin M. Barker, Christopher U. Meduri, Peter S. Fail, Jeffrey W. Chambers, Darrell J. Solet, Jacob M. Kriegel, Deborah C. Vela, Kari Feldt, Thomas D. Pate, Avni P. Patel, Tamaz Shaburishvili

**Affiliations:** a Section of Interventional Cardiology, Vanderbilt University Medical Center Nashville, Tennessee, USA; b Department of Cardiology, Karolinska University Hospital, Stockholm, Sweden; c Cardiovascular Institute of the South, Houma, Louisiana, USA; d Metropolitan Heart and Vascular Institute, Coon Rapids, Minnesota, USA; e Department of Surgery, Columbia University Irving Medical Center, New York, New York, USA; f Cardiovascular Pathology, Texas Heart Institute, Houston, Texas, USA; g Alleviant Medical, Austin, Texas, USA; h Tbilisi Heart and Vascular Clinic, Tbilisi, Georgia

**Keywords:** Heart failure, No-implant shunt, Preserved ejection fraction, Radiofrequency

## Abstract

**Background:**

Heart failure with preserved ejection fraction represents a major unmet clinical need with limited treatment options. Recent device therapies under investigation have focused on decompression of the left atrium through an implantable interatrial shunt. Although these devices have shown favorable safety and efficacy signals, an implant is required to maintain shunt patency, which may increase the patient risk profile and complicate subsequent interventions requiring transseptal access.

**Methods:**

The Alleviant System is a no-implant approach to creating an interatrial shunt using radiofrequency energy to securely capture, excise, and extract a precise disk of tissue from the interatrial septum. Acute preclinical studies in healthy swine (n = 5) demonstrated the feasibility of the Alleviant System to repeatably create a 7 mm interatrial orifice with minimal collateral thermal effect and minimal platelet and fibrin deposition observed histologically.

**Results:**

Chronic animal studies (n = 9) were carried out to 30- and 60-day time points and exhibited sustained shunt patency with histology demonstrating completely healed margins, endothelialization, and no trauma to adjacent atrial tissue. Preliminary clinical safety and feasibility were validated in a first-in-human study in patients with heart failure with preserved ejection fraction (n = 15). All patients demonstrated shunt patency by transesophageal echocardiographic imaging at 1, 3, and 6 months, as well as cardiac computed tomography imaging at 6-month follow-up timepoints.

**Conclusions:**

Combined, these data support the safety and feasibility of a novel no-implant approach to creating an interatrial shunt using the Alleviant System. Continued follow-up and subsequent clinical studies are currently ongoing.

## Highlights

• Patency of interatrial shunts via radiofrequency septectomy is maintained in swine and humans.
• Interatrial shunts created via radiofrequency septectomy heal with minimal thermal effect.
• A no-implant shunt method may be beneficial for treating congestive heart failure.

## Introduction

In the United States, more than 6.5 million people have heart failure, with 50% of patients classified as heart failure with preserved ejection fraction (HFpEF).^1^ Current treatment options for HFpEF are severely limited and consist mainly of diuretics for symptomatic improvement and focused treatment of hypertension and other comorbidities. Sodium-glucose cotransporter-2 inhibitors are an emerging treatment class that have recently demonstrated reduced heart failure hospitalization rates, but nonetheless, HFpEF remains a major clinical challenge.^2^ Device-based therapies for heart failure, including mechanical circulatory support and cardiac resynchronization therapy, are only indicated for heart failure with reduced ejection fraction. Currently, there are no Food and Drug Administration–approved device therapies that improve symptoms, reduce mortality, or reduce hospital admissions for patients with HFpEF.

Interatrial shunt (IAS) devices to treat HFpEF and heart failure with reduced ejection fraction are in various stages of design and clinical trials.^3^, ^4^, ^5^, ^6^, ^7^, ^8^, ^9^, ^10^, ^11^, ^12^, ^13^, ^14^, ^15^, ^16^, ^17^, ^18^, ^19^, ^20^ These devices leverage the principle of left atrial decompression via delivery of interatrial stents, or radiofrequency(RF)-ablative septostomy, to reduce left atrial filling pressures and relieve pulmonary vascular congestion. These therapies are being studied for their potential to reduce heart failure symptoms, improve quality of life, and reduce heart failure hospitalizations. Thus far, IASs have demonstrated positive safety signals and positive reported outcomes, including significant reduction in left ventricular filling pressures and improvements in 6-minute walk test, quality of life, and New York Heart Association classification.^6^^,^^8^^,^^12^^,^^13^^,^^15^ The REDUCE LAP-HF II trial (Corvia Medical, Tewksbury, Massachusetts) was a randomized, double-blinded, sham-controlled study comparing IAS therapy to placebo in 626 patients.^3^ Although the overall trial was neutral with respect to the composite endpoint of cardiovascular death, heart failure hospitalization, and quality of life improvement, subgroup analyses focusing on patients with exercise pulmonary vasculature resistance <1.74 Wood units suggest a significant potential benefit for shunt therapy.^21^

Despite promising efficacy and safety signals, implantable shunts come with clinical disadvantages. Reported complications associated with implantable shunts include device failure, malpositioning, embolization, and implant-associated thrombus.^8^^,^^18^^,^^22^ Additional challenges include increased procedural complexity, greater safety risks, limited options for future intracardiac interventions that require transseptal access, and technical difficulty of closing an implantable shunt should reversibility be desired. To mitigate the complexities and potential complications associated with intracardiac device implantation, a novel investigational device, the Alleviant System (Alleviant Medical, Inc, Austin, Texas), creates an IAS without requiring a permanent implant and is undergoing investigation in clinical trials. The device creates a 7 mm shunt via controlled RF excision and removal of a segment of the atrial septum. In the absence of a septal implant, it is critical to characterize the tissue-response properties and long-term patency data of this no-implant approach to interatrial shunting. Here, we report the preclinical results of the Alleviant System, focusing on device performance and associated histologic outcomes in a porcine model, as well as human shunt patency data in the first 15 patients treated through a pilot clinical study with 6-month follow-up.

## Methods

### Device Description

**Figure 1:**
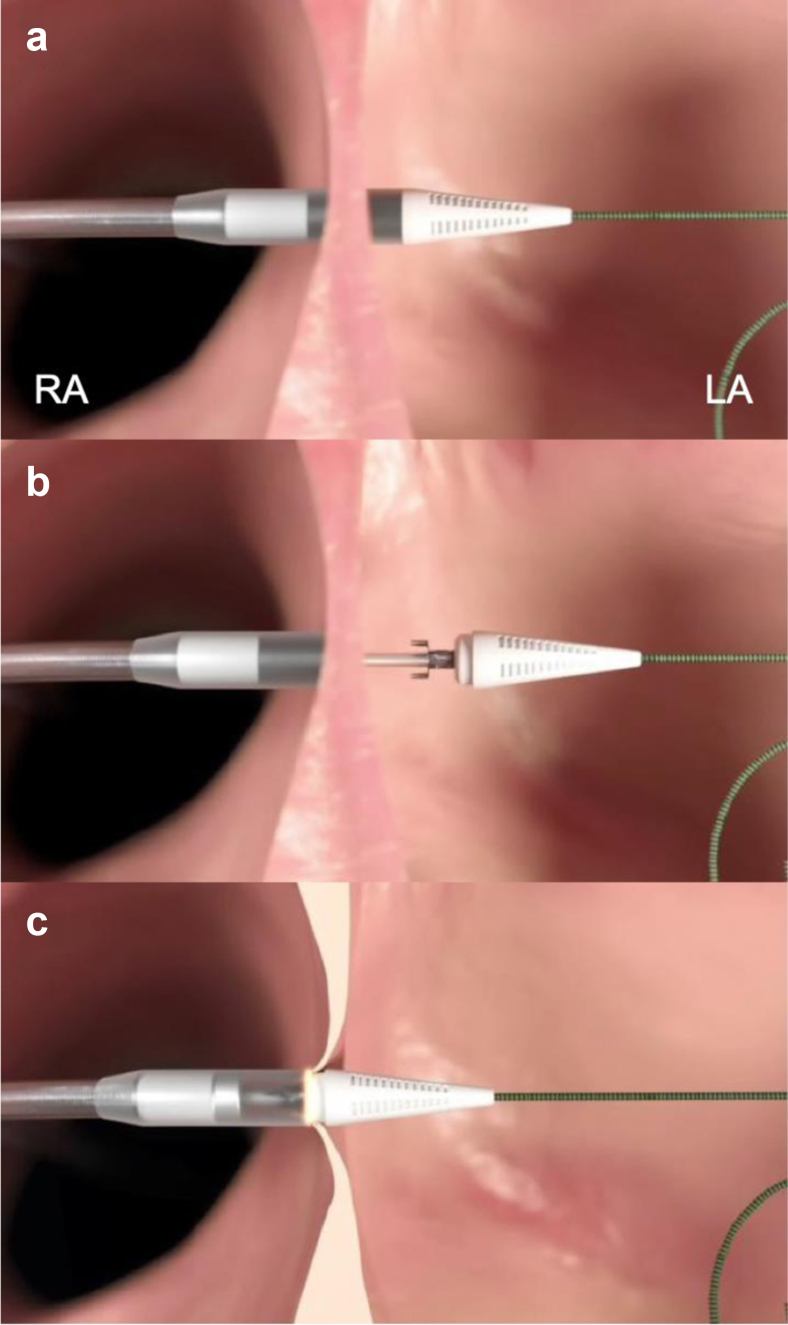
**Overview of the Alleviant procedure.** (a) The tip of the device is advanced across the interatrial septum. (b) The tip of the device is opened to deploy the tissue anchoring mechanism within the LA. (c) The tip of the device is closed, thereby securing the target tissue, and a pulse of radiofrequency energy is applied to excise the tissue. Abbreviations: LA, left atrium; RA, right atrium.

The Alleviant System comprises a single-use catheter and electrosurgical generator. The catheter is 16 Fr and is intended for transfemoral deployment after standard transeptal puncture and wire delivery to the left atrium. After tip deployment and coaptation onto the interatrial septum, the catheter creates a 7 mm diameter orifice in the fossa ovalis via electrosurgical excision of interatrial tissue via short pulse energy delivered by the generator (Figure 1). The target septal tissue is securely captured within the device before excision and is retained until the device is removed from the body. Controlled excision of atrial septal tissue using RF energy enables the creation of a precise and durable atrial shunt with no required cardiac implant.

Given the novel mechanism of the IAS created electrosurgically by the Alleviant System, a preclinical evaluation of its performance and associated histologic outcomes after shunt creation was conducted in a porcine model (n = 13) across multiple timepoints (acute [<1 ​hour], 30 days, and 60 days postprocedure). Shunt creation and durability were also examined in a human feasibility study (n = 15) with imaging at baseline and 6 months.

### Preclinical Evaluation

The Alleviant procedure was performed on 13 healthy male domestic Yorkshire or Hampshire swine (66.2-81.8 kg) at the Houston Methodist Research Institute (PCCL, Houston, Texas) and American Preclinical Services (Minneapolis, Minnesota). The studies included acute and chronic survival (t = 30 days and 60 days) cases. All procedures used the same interventional methods, and study protocols were approved by the corresponding Institutional Animal Care and Use Committees for compliance with regulations and accepted practices.

### Operative Procedure

All animals studied acutely received daily dual antiplatelet therapy (DAPT) regimen of 81 mg aspirin and 75 mg clopidogrel, initiated 3 days before the procedure. For chronic survival cases, DAPT was initiated 7 days preprocedure. Procedures were performed under general anesthesia. Procedural steps were similar for all groups. The first step included placement of introducer sheaths through the right and left femoral veins to allow device access (16 Fr) and for intracardiac echocardiography. Under fluoroscopic and echocardiographic image guidance, a standard transseptal puncture was performed through the fossa ovalis, and an 0.035” guidewire was delivered to the left atrium. The device was introduced and advanced across the septum at the fossa ovalis. After confirmation of device positioning, the distal tip of the device was deployed and coapted onto the septum, capturing and securing the targeted tissue inside of the catheter. A short pulse of RF energy was then applied to circumferentially excise the captured septal tissue, thereby creating an IAS. The device, with excised tissue captured within, was then removed and inspected *ex vivo* to confirm tissue capture. Shunt creation was confirmed using intracardiac echocardiography color Doppler imaging for measurement of shunt size and visualization of shunt flow from the left atrium to the right atrium in all cases.

### Postsacrifice Examination and Histology

After humane euthanization, the porcine hearts were explanted and photographed, and a gross assessment was performed on the IAS and surrounding cardiac tissue. The excised hearts were then immersed in 10% neutral-buffered formalin for further macroscopic and microscopic examination. Three to five representative samples of the interatrial septum were processed for paraffin embedding, sectioned at 5 μm thickness, and stained with hematoxylin and eosin and Movat pentachrome. Histopathologic analyses were performed on each slide section by light microscopy and included semiquantitative assessment of the shunt margin, including inflammation, endothelialization, hemorrhage, collagen denaturation, coagulative necrosis, fibrosis, mineralization, and thrombus.

### Preliminary Clinical Development

The Alleviant System has been evaluated in a first-in-human (FIH) study, ALLEVIATE-HF-1, as a single-center, prospective, open-label trial in 15 patients treated at the Tbilisi Heart and Vascular Clinic (Tbilisi, Georgia). The study protocol, informed consent form, Investigator Brochure, and Instructions for Use were reviewed and approved by the local ethics committee. All patients provided informed consent before enrollment. The trial was registered at clinicaltrials.gov (NCT04583527).

The primary objective of the trial was to establish the safety and feasibility of the Alleviant System for the creation of a durable no-implant IAS in eligible HFpEF patients. Shunt patency was assessed via transthoracic echocardiography performed at 1-, 3-, and 6-month follow-up, through transesophageal echocardiography (TEE) performed intraprocedurally and at 6-month follow-up, and through cardiac computed tomography (CT) performed at discharge and 6-month follow-up.

## Results

The Alleviant procedure was successfully performed in all 13 preclinical and all 15 clinical procedures with confirmation of shunt creation via intracardiac echo and through verification of excised septal tissue contained within the device. No intraprocedural adverse events were observed. All shunts appeared circular and well formed in the fossa ovalis with no adverse findings observed under echo.

### Acute Treatment Group

**Figure 2:**
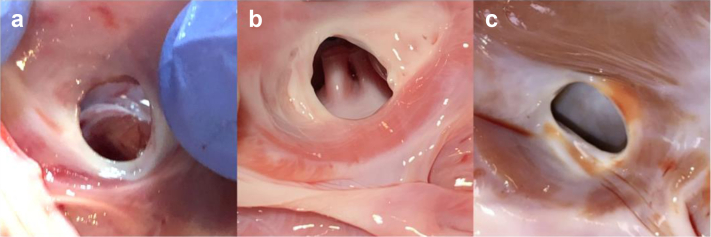
**Representative images of fresh interatrial shunt situated within the boundaries of the fossa ovalis across all timepoints (right atrial view).** (a) Acute specimen shows the orifice edges appear rounded and expose the acutely sectioned tissue. (b) Thirty-day chronic shows surfaces of the shunt orifice edge and neighboring tissue appear smooth and glistening. (c) Sixty-day chronic shows a circular to mildly oval orifice with smooth edges.

**Figure 3:**
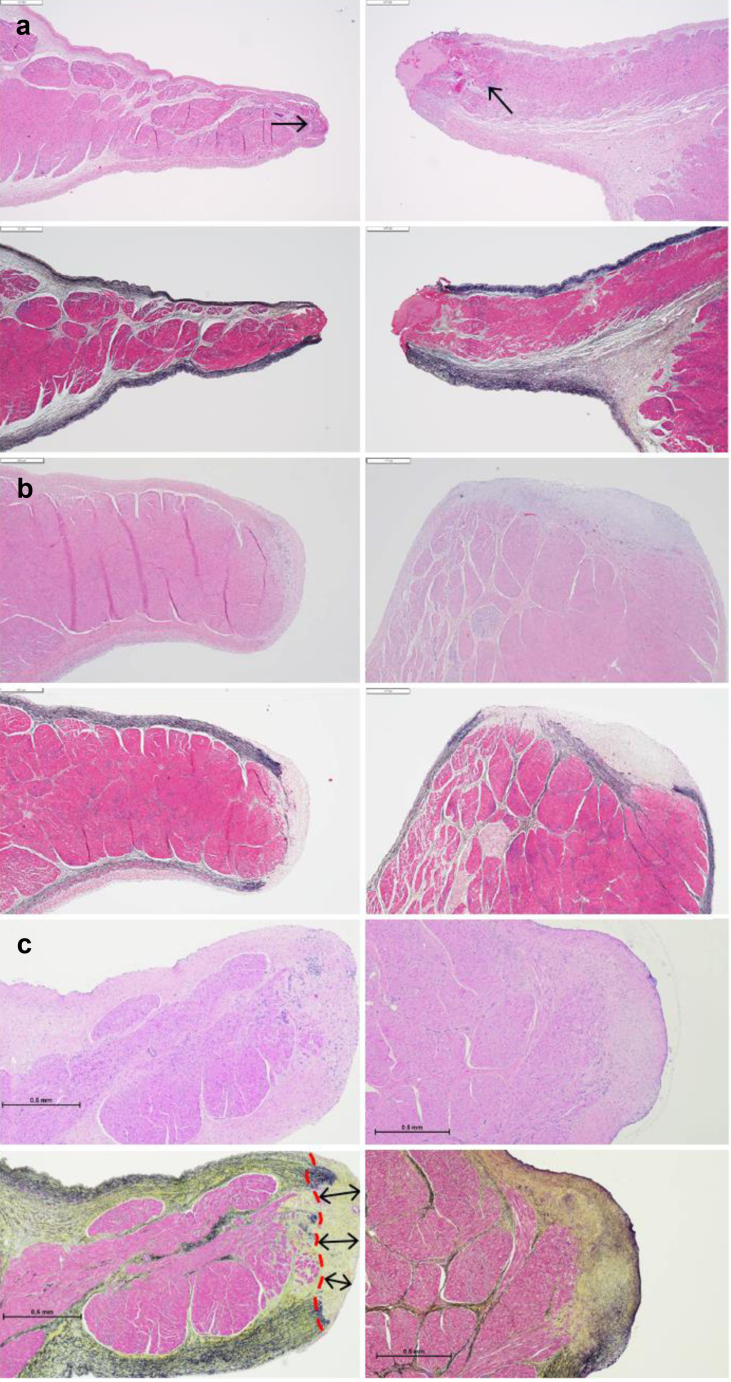
**Representative histologic sections across all timepoints.** (a) Acutely cut posterior (left panel) and anterior (right panel) shunt edges present a tapered, rounded contour, with modest platelet/fibrin deposition in the anterior edge. A very thin band of coagulative changes (thermal effect) is also present in the myocardium adjacent to the cut (arrows, approximately 400 μm in depth). (b) Thirty-day shunt edges appeared rounded and fully healed, displaying fibrous remodeling and endothelialization. (c) Sixty-day sample (Movat) shows approximately 350 μm of collagen deposition at the rounded shunt edge, indicative of remodeling and normal healing. Stains: hematoxylin and eosin (top) and Movat (bottom); bar = 500 μm.

Animals in the acute treatment group were sacrificed following the procedure. Macroscopically, all animals presented with a widely patent shunt that was round (Figure 2a). Shunt diameter on the explanted hearts was measured to be 6.8 ± 0.9 mm. Microscopic analysis indicated a minimal layer of platelet/fibrin deposition where the device cut through the interatrial wall. All acute animals showed minimal focal coagulative changes and occasional mild interstitial hemorrhage that was <500 μm thick on average (Figure 3a; range 375-600 μm). The tissue beyond this rim was normal, indicating minimal postoperative injury.

### Chronic Treatment Group

Animals in the chronic treatment groups were sacrificed at various timepoints as summarized in Table 1. Macroscopically, all animals demonstrated a widely patent shunt in the interatrial septum that was round or oval with well-healed, smooth, and rounded edges (Figure 2b and c). Microscopic evaluation of specimens from animals sacrificed at 30 and 60 days demonstrated edges of the shunt margin that were completely healed with fibrous remodeling (Figure 3b and c). The edges consisted of fibrous connective tissue, with complete endothelialization at the surface and smooth transition to the adjacent atrial tissue.

**Table 1:** Preclinical summary (swine model)

No.	Study type	Study duration	Weight (kg)	Patent shunt
01	Acute	--	66.2	Yes
02	Acute	--	72.5	Yes
03	Acute	--	73.0	Yes
04	Acute	--	76.6	Yes
05	Acute	--	73.0	Yes
06	Chronic	30 d	74.0	Yes
07	Chronic	30 d	70.0	Yes
08	GLP chronic	60 d	78.4	Yes
09	GLP chronic	60 d	68.2	Yes
10	GLP chronic	60 d	74.2	Yes
11	GLP chronic	60 d	81.8	Yes
12	GLP chronic	60 d	74.2	Yes
13	GLP chronic	60 d	77.0	Yes

GLP, good laboratory practice.

### ALLEVIATE-HF-1 FIH Pilot Study

**Figure 4:**
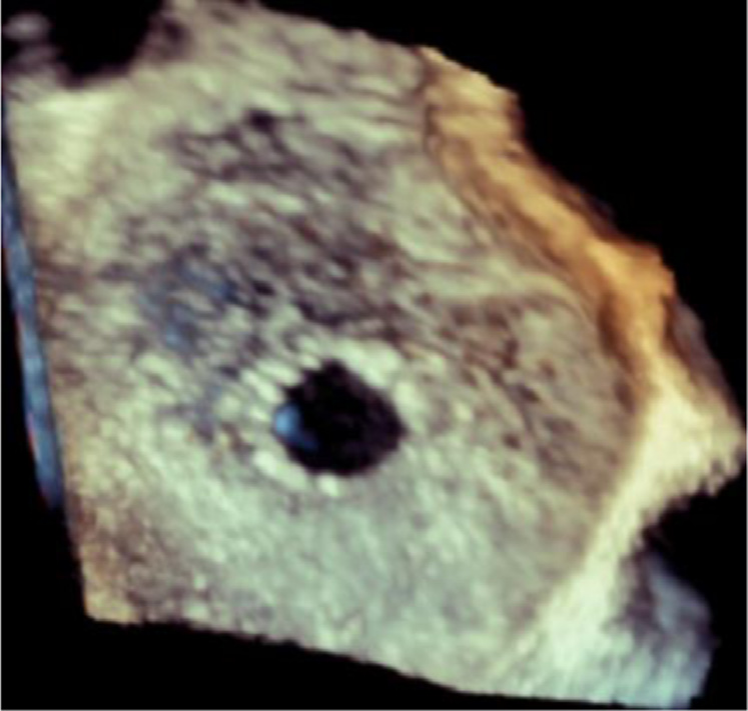
Representative images of 3D transesophageal echocardiographic assessment of interatrial shunt immediately following shunt creation in a human subject.

**Figure 5:**
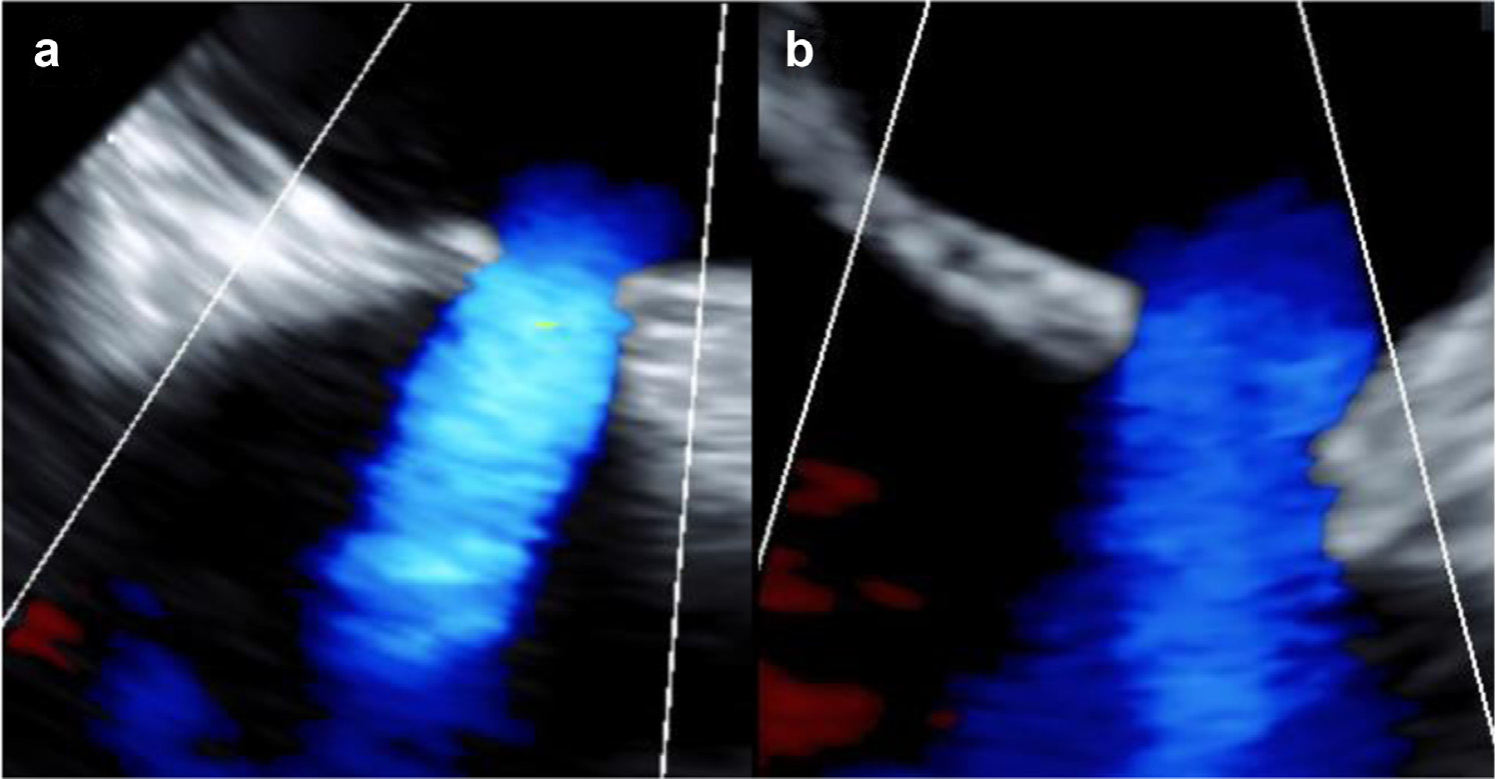
**Representative images of transesophageal echocardiographic assessment of a human subject showing shunt patency sustained through 6-month follow-up.** (a) Baseline, immediately post-shunt creation. (b) Six-month follow-up.

**Figure 6:**
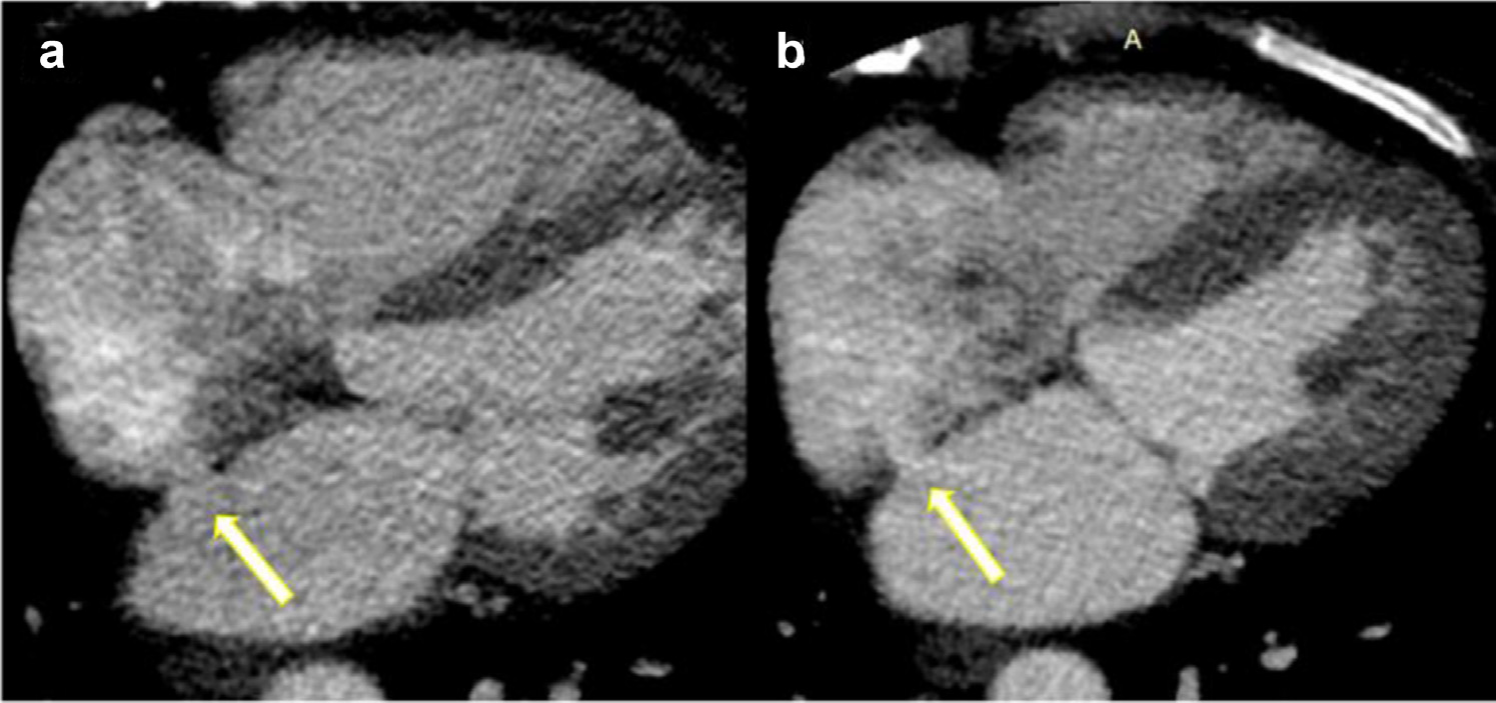
**Representative images of cardiac computed tomography assessment showing shunt patency sustained through 6-month follow-up in a human subject.** (a) Baseline, immediately postshunt creation. (b) Six-month follow-up.

Successful tissue excision and IAS creation were achieved in all 15 patients, and the mean shunt size at baseline was 7.0 ± 0.9 mm as measured via TEE (color Doppler) at the time of procedure. Figure 4 shows a representative image of the IAS at baseline (3D TEE). At 6-month follow-up, all shunts remained patent with left to right flow by TEE (Figure 5) or by CT imaging demonstrating an interatrial communication (Figure 6). A more detailed report of this FIH cohort, including baseline characteristics, clinical outcomes, and shunt size characterization, is forthcoming.

## Discussion

Preclinical evaluation of the Alleviant System demonstrated clean shunt margins, both acutely and chronically, with shunt patency maintained through follow-up for all animals (up to 60 days). Histologic analysis showed smoothly healed shunt edges with uniform endothelialization and limited depth of injury (<500 μm on average). The microscopic findings were indicative of a stable healing response to a small injury. Although subsequent time points beyond 60 days were not investigated in this preclinical study, the fibrous remodeling observed at shunt margins and absence of inflammation in all 60-day chronic animals evaluated is suggestive of a mature healing response and may point to sustained long-term shunt stability. Furthermore, as mentioned previously, shunt patency has been confirmed in all 15 human patients at 6-month follow-up by TEE and/or CT.

Early clinical evidence supports the therapeutic potential of IAS for the treatment of congestive heart failure, and multiple implantable shunts are under study in US pivotal trials. Although intracardiac device implantation has shown success in early clinical trials, it comes with additional risks inherent to implants, including device-associated thrombus, dislodgement, and device failure.^22^^,^^23^ Although patency is expected with implantable shunts, there have been instances of device occlusion, rendering it a treatment with variable outcomes.^18^^,^^20^ Delivering the potential therapeutic benefits of an IAS without a permanent cardiac implant may have clinical and procedural advantages. These include avoidance of device embolization, tissue erosion, device-associated thrombus formation, procedure-related device placement complications, the need for long-term DAPT, and preservation of native septal tissue for future transseptal access. The Alleviant approach avoids complications associated with implants via a device that captures the target tissue for excision and applies mechanical compression before applying RF energy, thereby controlling the extent of the tissue excised as well as minimizing the endothelial injury incurred to surrounding tissue. A 30-day DAPT regimen is recommended postprocedure to minimize the aggregation of platelets at the shunt margin. In addition, the inherent left-to-right pressure gradient present in HFpEF patients provides a consistent flow of blood across the shunt, further promoting shunt patency. Human studies to assess the clinical performance of the Alleviant IAS device, including long-term shunt patency, are currently underway, and a US pivotal study is planned for 2022.

There are several additional elements to the Alleviant approach that are believed to be critical to ensuring the creation of a durable IAS. The electrosurgical excision of tissue from the interatrial septum is unique from current methods for the creation of a defect in the septum, for example, balloon atrial septostomy, which simply tear or stretch tissue under a rapid pull-force rather than excise tissue. Such approaches are limited in their ability to control the size of the defect created and also carry the risk of spontaneous or premature closure due to overlapping of the edges over time. These characteristics limit their utility for the treatment of heart failure. In contrast, removal of tissue from the interatrial septum results in a shunt that maintains long-term patency. This insight comes from the Blalock-Hanlon operation, in which a portion of the interatrial septum is surgically removed during an open-heart operation. In early articles describing this operation, the authors noted that the shunt remained permanently patent after tissue excision and that on pathologic analysis, “the margins were always well healed and smooth.”^24^^,^^25^ This open surgical experience provides supporting evidence that a defect will remain patent on the excision of tissue. With the exception of a small series published by Blalock et al. from 1948 to 1950, little is known about the healing response of the atrial septum after excision of tissue.^26^, ^27^, ^28^, ^29^ Preclinical evaluation of electrosurgical IAS creation by the Alleviant System in the present study and associated histologic outcomes confirms the sustained patency of septal excision and gives new insights into the healing response of the atrial septectomy.

Although the porcine model has similar anatomy and physiology to the human cardiovascular system, this study does have limitations related to the model. The most notable limitation of the preclinical study is the absence of heart failure and the associated absence of a left-to-right atrial pressure gradient. Other limitations include anatomical differences (smaller septum, fossa ovalis, and left atrium, thus requiring a tighter angle of approach for perpendicular device positioning), greater sensitivity to atrial fibrillation and arrhythmia, and altered pulmonary vein anatomical locations.

## Conclusions

There are no Food and Drug Administration–approved devices for the creation of an IAS as a therapeutic modality for heart failure. The Alleviant System was designed to create a durable IAS and has been evaluated through preclinical animal studies and human feasibility studies as reported here. Preclinical testing with the Alleviant System showed minimal thermal effect, sustained shunt patency, and evidence of a mature healing response in the chronic animals. First-in-human clinical evaluation of the Alleviant device demonstrated that patients treated with implant-free shunts maintained shunt patency through 6 months. The novel Alleviant device mechanism enables controlled atrial shunt creation that may lead to a long-term patent IAS. As more data emerge supporting the role of IAS for heart failure, elucidation of the healing response of nonimplantable shunts will be critical to understanding their long-term performance.

## Ethics Statement

All research reported has adhered to the relevant ethical guidelines: Study protocols for the preclinical evaluation were approved by the corresponding Institutional Animal Care and Use Committees for compliance with regulations and accepted practices. For the first-in-human study, the study protocol, informed consent form, Investigator Brochure, and Instructions for Use were reviewed and approved by the local ethics committee and all patients provided informed consent before enrollment.

## Funding

These studies were funded by Alleviant Medical. Preclinical research reported in this publication was supported by the 10.13039/100000050National Heart, Lung, And Blood Institute of the 10.13039/100000002National Institutes of Health under Award Number R43HL142440 and R44HL142440. The content is solely the responsibility of the authors and does not necessarily represent the official view of the National Institutes of Health.

## Disclosure statement

C. M. Barker reports consulting fees from and is an advisory board/board member to Alleviant Medical. C. U. Meduri reports consulting fees from Alleviant Medical, Anteris Technologies, Boston Scientific, Medtronic, Vdyne, speakers’ fees from Abbott, Boston Scientific, Edwards Lifesciences, and Medtronic; he is an advisory board member for Anteris Technologies and Cardiovalve, and a proctor for Boston Scientific. P. S. Fail reports consulting fees from BioVentrix and Alleviant Medical, speakers’ fees from Abbott Vascular, Boston Scientific, and Medtronic, and served as principal investigator of research studies for Ancora Heart and Corvia Medical. J. W. Chambers reports consulting fees from Alleviant Medical and serves as Chief Medical Officer of Cardiovascular Systems Inc. D. J. Solet reports consulting fees from Abbott Medical and Alleviant Medical. J. M. Kriegel reports consulting fees from, is an advisory board/board member to, and serves as Chief Medical Officer of Alleviant Medical. K. Feldt reports consulting fees from Abbott Vascular, Alleviant Medical, Anteris Technologies, Pfizer Inc, and Orion Pharma. T. D. Pate and A. P. Patel are employed by Alleviant Medical. T. Shaburishvili served as principal investigator of a research study for Alleviant Medical. All other authors declare no competing interests.
